# Single-Cell Transcriptomics Reveal a Correlation between Genome Architecture and Gene Family Evolution in Ciliates

**DOI:** 10.1128/mBio.02524-19

**Published:** 2019-12-24

**Authors:** Ying Yan, Xyrus X. Maurer-Alcalá, Rob Knight, Sergei L. Kosakovsky Pond, Laura A. Katz

**Affiliations:** a Smith College, Department of Biological Sciences, Northampton, Massachusetts, USA; b University of Massachusetts Amherst, Program in Organismic and Evolutionary Biology, Amherst, Massachusetts, USA; c University of California San Diego, Department of Pediatrics, San Diego, California, USA; d University of California San Diego, Department of Computer Science and Engineering, San Diego, California, USA; e University of California San Diego, Center for Microbiome Innovation, San Diego, California, USA; f Temple University, Institute for Genomics and Evolutionary Medicine, Philadelphia, Pennsylvania, USA; Harvard University

**Keywords:** transcriptomics, gene family evolution, genetic code evolution, phylogenomics, Ciliophora, uncultivable microbes

## Abstract

Ciliates, a eukaryotic clade that is over 1 billion years old, are defined by division of genome function between transcriptionally inactive germline micronuclei and functional somatic macronuclei. To date, most analyses of gene family evolution have been limited to cultivable model lineages (e.g., *Tetrahymena*, *Paramecium*, *Oxytricha*, and *Stylonychia*). Here, we focus on the uncultivable Karyorelictea and its understudied sister class Heterotrichea, which represent two extremes in genome architecture. Somatic macronuclei within the Karyorelictea are described as nearly diploid, while the Heterotrichea have hyperpolyploid somatic genomes. Previous analyses indicate that genome architecture impacts ciliate gene family evolution as the most diverse and largest gene families are found in lineages with extensively processed somatic genomes (i.e., possessing thousands of gene-sized chromosomes). To further assess ciliate gene family evolution, we analyzed 43 single-cell transcriptomes from 33 ciliate species representing 10 classes. Focusing on conserved eukaryotic genes, we use estimates of transcript diversity as a proxy for the number of paralogs in gene families among four focal clades: Karyorelictea, Heterotrichea, extensive fragmenters (with gene-size somatic chromosomes), and non-extensive fragmenters (with more traditional somatic chromosomes), the latter two within the subphylum Intramacronucleata. Our results show that (i) the Karyorelictea have the lowest average transcript diversity, while Heterotrichea are highest among the four groups; (ii) proteins in Karyorelictea are under the highest functional constraints, and the patterns of selection in ciliates may reflect genome architecture; and (iii) stop codon reassignments vary among members of the Heterotrichea and Spirotrichea but are conserved in other classes.

## Abstract (precis)

Ciliates, a eukaryotic clade that is over 1 billion years old, are defined by division of genome function between transcriptionally inactive germline micronuclei and functional somatic macronuclei. To date, most analyses of gene family evolution have been limited to cultivable model lineages (e.g., *Tetrahymena*, *Paramecium*, *Oxytricha*, and *Stylonychia*). Here, we focus on the uncultivable Karyorelictea and its understudied sister class Heterotrichea, which represent two extremes in genome architecture.

## INTRODUCTION

Most work on genome evolution in ciliates has focused on a few cultivable model lineages (e.g., *Tetrahymena* and *Paramecium*) that represent only a small proportion of biodiversity within this ancient (∼1 billion-year-old) clade. In the present study, we analyze data from a diverse sample of uncultivable ciliates, particularly the class Karyorelictea, which has very few published molecular data, that we isolated by hand from diverse environments.

Single-cell transcriptomics (SCT) have yielded insights in diverse fields, including microbial ecology, neurobiology, stem cell research, and cancer research (1–3). Developed in 2009 for analyses of blastomere transcriptomes in mice, SCT has since been used in a large number of studies focusing on microbes, primarily on bacteria (4, 5). Single-cell transcriptome techniques were first applied to ciliates, the focus of the present study, by Kolisko et al. (6), who reported that data generated from single-cell transcriptomics recovered approximately 90% of transcripts found from traditional total RNA extraction of stable cultures. Unsurprisingly, the number of assembled transcriptomes in SCT experiments varies with cell size and among individuals within species, the latter likely due to differences in life history stages (6, 7). Nevertheless, a major strength of single-cell transcriptomics is the recovery of gene sequences from uncultivable lineages, which constitute the majority of microbial eukaryotes.

Ciliates are a group of microbial eukaryotes that have somatic and germline genomes in separate nuclei sharing a common cytoplasm. In ciliates, somatic macronuclei are transcriptionally active and possess an atypical genome architecture: somatic chromosomes are often gene-dense, lack centromeres, exist at high copy number (∼45 N in Tetrahymena thermophila and ∼15,000 N in *Stylonychia lemnae* [8–10]), and in some lineages, are extensively fragmented to generate gene-sized somatic chromosomes (e.g., ∼2.2 kbp on average in *S. lemnae* [11]). In all but one class of ciliates, the class Karyorelictea, these processed somatic nuclei divide by amitosis, a noncanonical form of nuclear division (i.e., lacks clear spindles and without clear chromosome condensation) that divides the polyploid somatic macronuclei (12). The germline genome remains quiescent throughout asexual cycles, becoming transcriptionally active only during the sexual phases. Unlike the somatic genome, the germline chromosomes are genomically conventional (i.e., they possess centromeres and are several megabases long [10, 13]).

A challenge for interpreting microbial transcriptome data is the use of alternative genetic codes (14–16) since many ciliates, and an increasing number of other eukaryotic lineages, have been shown to reassign one or more canonical stop codons to various amino acids (17–20). In ciliates, genetic codes tend to fall into one of three classes: (i) standard (UAA, UAG, and UGA) stop codons are used for translation termination (i.e., canonical “universal” genetic code; e.g., *Dileptus* [21] [Cl: Litostomatea], *Nyctotherus* [22] [Cl: Armophorea], and *Stentor* [23] [Cl: Heterotrichea]); (ii) UAG and UAA are recognized as translation termination signals, with UGA coding for cysteine or tryptophan, e.g., *Euplotes* (24) (Cl: Spirotrichea) and *Blepharisma japonicum* (15) (Cl: Heterotrichea), respectively; and (iii) UGA is the sole functional stop codon, whereas UAA and UAG are translated into glutamine (e.g., *Tetrahymena* [25], *Paramecium* [26] [Cl: Oligohymenophorea], *Oxytricha* and *Stylonychia* [14] [Cl: Spirotrichea]), tyrosine (*Mesodinium rubrum* [15]), or glutamic acid (*Campanella umbellaria* [15] [Cl: Oligohymenophorea]). Even more unusual, *Condylostoma magnum* (15, 16) (Cl: Heterotrichea) follows none of the three strategies and reassigns all three standard stop codons to sense codons; in this lineage, interpreting the function of stop codons depends on their context in the mRNA (i.e., proximity to the 3′ untranslated region (UTR) and poly(A) tail [16]).

In addition, the majority of the species from which we isolated and collected transcriptome data lack reference genomes/transcriptomes even from closely related taxa. Thus, contamination removal, open reading frame (ORF) prediction, and gene family assignment from *de novo* transcriptome assembly is another major challenge. In the present study, we rely on PhyloToL (27), a taxon- and gene-rich bioinformatic pipeline that has been successfully used to analyze high-throughput sequencing (HTS) data from diverse eukaryotes (27). PhyloToL allows for a conservative approach addressing bioinformatic bleeding, contamination, and sequencing/assembly errors associated with HTS data.

Previous work has linked genome architecture to gene family evolution in ciliates (28). First, protein coding genes in ciliates tend to evolve faster than in other eukaryotes (29, 30). Second, ciliates with extensively fragmented somatic genomes (i.e., gene-sized somatic chromosomes) have more of and more diverse paralogs compared to ciliates without extensively fragmented somatic chromosomes (31, 32). However, these observations are mostly limited to taxa from the large Intramacronucleata clade (referred to here as the Im-clade), particularly the model ciliates *Tetrahymena*, *Paramecium*, and *Oxytricha*. Moreover, many analyses of gene family evolution have focused on only a few conserved genes, such as actin, α-tubulin, HSP90, dynein heavy chain family (32–36), and lineage specific genes, such as pheromones in *Euplotes* (37). Gene family evolution in other genes and across the other major ciliate subphylum Postciliodesmatophora (referred to as the Po-clade), containing the classes Karyorelictea and Heterotrichea, remains poorly understood.

To expand our knowledge of ciliate genome evolution, we sampled uncultivable ciliates, focusing on the understudied classes Karyorelictea and Heterotrichea (Po-clade) that have distinct genome features. The somatic nuclei in all Karyorelictea are described as paradiploid (i.e., have similar DNA content to the germline nuclei), lack the ability to divide by amitosis, and are differentiated from germline nuclei during both asexual and sexual cycles (38). In contrast, Heterotrichea, the sister clade to the Karyorelictea, contain highly amplified somatic genomes (e.g., ∼1,000 to ∼13,000 times more DNA compared to germline nuclei [39]) that are often housed in large nuclei resembling a chain of beads, and the somatic macronucleus is capable of amitosis, dividing with extramacronuclear microtubules (versus intramacronuclear microtubules for members in Im-clade) (10). Thus, even though Karyorelictea and Heterotrichea group together as sister clades, they represent strikingly different nuclear characteristics.

Here, we use single-cell transcriptome analyses of uncultivable ciliates to investigate the impact of these variable somatic genome structures on patterns of gene family evolution. We characterize transcripts from 43 individuals representing 33 species and 10 classes, including 11 species of Karyorelictea and 6 species of Heterotrichea focusing on transcript diversity, transcript divergence, and stop codon reassignments across the ciliate phylogeny. Using bioinformatics tools such as PhyloToL (27) and RELAX (40), we analyze 509 genes to assess the relationship between patterns of molecular evolution and genome architecture.

## RESULTS

### Single-cell transcriptomes.

We collected single-cell transcriptomic data from 43 individuals representing 33 species from ten ciliate classes, focusing on the poorly studied classes Karyorelictea and Heterotrichea (Table 1), and we combined these data with 13 transcriptomes available from public databases (see Table S3 in the supplemental material). After using PhyloToL (27; https://github.com/Katzlab/PhyloTOL) to remove rRNA sequences and potential prokaryotic contaminants, our single-cell transcriptomes yielded an average of 3,278 (range, 213 to 12,012) transcripts falling into 1,665 (range, 159 to 3,894) distinct gene families (GF; Table S1). For the newly generated data, we combined transcriptomes from individuals belonging to the same species (as determined by shared small subunit-rDNA sequences) for analyses of GF evolution (see Materials and Methods for details).

**TABLE 1:** Summary of ciliate single-cell transcriptomes included in the present work, focusing on diverse species from ten classes of ciliates^*a*^

Focal clade	Class	No. of genera	No. of species	WTA	Species
Karyorelictea	Karyorelictea	7	11	14	*Cryptopharynx* sp., *Geleia acuta*, *Geleia sinica*, *Geleia* sp., *Kentrophoros* sp., *Loxodes* sp. 1, *Loxodes* sp. 2, *Loxodes striatus*, *Remanella* sp., Trachelocercidae sp. 1, Trachelocercidae sp. 2
Heterotrichea	Heterotrichea	5	6	8	*Anigsteinia* sp., *Blepharisma americanum*, *Climacostomum* sp., *Spirostomum ambiguum*, *Spirostomum minus*, *Stentor roeselii*
Extensive fragmenters	Armophorea	2	2	2	*Brachonella spiralis*, *Metopus* sp.
	Phyllopharyngea	1	1	2	*Chilodonella uncinata*
Non-extensive fragmenters	Colpodea	1	1	2	*Bursaria truncatella*
	Litostomatea	4	4	6	*Didinium nasutum*, *Rimaleptus mucronatus*, *Litonotus* sp., *Spathidium* sp.
	Nassophorea	1	1	2	*Zosterodasys* sp.
	Oligohymenophorea	3	3	3	*Frontonia* sp., *Lembadion* sp., *Vorticella* sp.
	Plagiopylea	2	2	2	*Parasonderia* sp., *Sonderia* sp.
	Prostomatea	2	2	2	*Prorodon ovum*, *Nolandia orientalis*

a WTA, whole transcriptome amplification (for further details, see Table S1 in the supplemental material). Although limited evidence of fragmentation in somatic genome has been reported in Litostomatea (51), we have assigned this class to the non-extensive fragmenters while awaiting further data.

10.1128/mBio.02524-19.2 TABLE S1 Detailed information of the ciliate single-cell transcriptomes included in the present work. GF, gene family. Average and median K-mer coverage per transcript are assessed after combining individuals from same species. Related to sampling in Materials and Methods. Download Table S1, XLSX file, 0.02 MB. Copyright © 2019 Yan et al. 2019 Yan et al. This content is distributed under the terms of the Creative Commons Attribution 4.0 International license.

10.1128/mBio.02524-19.4 TABLE S3 Ciliate transcriptomes that are obtained from public databases. Related to transcript diversity analyses in Materials and Methods. Download Table S3, XLSX file, 0.01 MB. Copyright © 2019 Yan et al. 2019 Yan et al. This content is distributed under the terms of the Creative Commons Attribution 4.0 International license.

**FIG 1:**
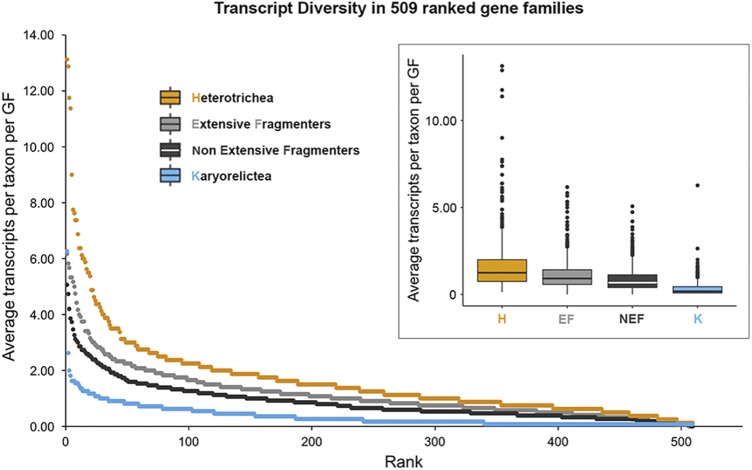
Rank curve and boxplot showing lower average transcript diversity of Karyorelictea (blue), compared to extensive fragmenters (EF, light gray), non-extensive fragmenters (NEF, dark gray), and Heterotrichea (yellow), in analyses of 509 gene families. All pairwise comparisons are significant (*P* < 0.05, Kruskal-Wallis nonparametric test). GF, gene family.

For comparison, we separate the Im-clade into two non-monophyletic groups, those with extensively fragmented somatic genomes (EF; Armophorea, Spirotrichea, and Phyllopharyngea; 12 species, Table 1 and Fig. 1) and those with putative non-extensively fragmented genomes (NEF; 16 species, Table 1 and Fig. 1). This allows us to evaluate the impact of extensively fragmented somatic genomes, which are known to contribute to GF expansion (31, 41). For all subsequent analyses, we focus on the 509 conserved eukaryotic GFs present in at least 1 member of all four focal clades: Karyorelictea, Heterotrichea, EF, and NEF.

### Transcript diversity in ciliates.

To evaluate patterns of GF evolution across ciliates, we estimated the transcript diversity per GF for each taxon in our data set and then assessed the patterns within four groups: Karyorelictea, Heterotrichea, EF, and NEF. Among the 509 gene families included in the analyses, the average transcript diversity in Karyorelictea is the lowest among the four focal clades (Fig. 1). Among the four groups, we observe the following order of average transcript diversity per GF per taxon: Heterotrichea > EF > NEF > Karyorelictea (1.25, 0.92, 0.67, and 0.18, respectively). In over half of all GFs (319/509; 62.67%), each karyorelictid species included in our analyses possessed a single transcript. Similarly, the median number of transcripts per GF (i.e., paralogs) among the Karyorelictea is significantly lower than those of Heterotrichea, EF, and NEF (*P* < 0.001, Kruskal-Wallis two-sided test). In Karyorelictea, the variability in average number of transcripts per GF is also lower compared to the other three groups (interquartile range/median: Karyorelictea = 0.36, Heterotrichea = 1.25, EF = 0.84; NEF = 0.73; Fig. 1). To our surprise, the average transcript diversity of Heterotrichea is the greatest (*P << *0.001, Kruskal-Wallis two-sided test) and most variable among the four groups, indicating greater paralog diversity of highly expressed genes.

### Selection analysis.

We also investigated the strength of selection acting on gene families in our four focal groups using RELAX (42), assessing intensification versus relaxation based on selection intensity parameter (*K*) with the NEF chosen as the reference group. In nearly half of the alignments tested (236/503 or 46.9%, Table 1; see also Table S5 in the supplemental material) we were able to detect differences (*P* < 0.05) in selective strengths between the four focal groups with the RELAX test (Table 1; see also Table S5). The direction of change (intensification versus relaxation relative to NEF) was evenly split among alignments for EF branches (120/236, 116/236). However, for both Heterotrichea (179/236, 57/236) and Karyorelictea (148/236, 88/236; Table 1; see also Table S5), branches evolved under stronger selection (compared to NEF) more frequently than under weaker selection. Because intensification of selection could be a consequence of either stronger negative selection (here we use ω to represent the *dN/dS* ratio [i.e., the ratio of nonsynonymous to synonymous evolutionary changes (or substitutions)]; lower ω for ω < 1) and/or stronger positive selection (higher ω for ω > 1), we further examined patterns of ω variation across groups. Specifically, we computed means of ω values conditioned on ω < 1 (i.e., the negatively selected component of the distribution) based on the fits from the partitioned exploratory RELAX models (Fig. S1). Based on previous observations on the relationship between genome architecture and patterns of molecular evolution (31, 32, 43), we tested the *a priori* ordering of groups: Karyorelictea < NEF < Heterotrichea < EF and found a statistically significant trend (*P* < 0.001, Jonckheere-Terpstra test). In other words, our inferences are consistent with stronger functional constraints in the Karyorelictea compared to either reduced constraints and/or positive selection operating on lineages in the non-monophyletic EF group.

10.1128/mBio.02524-19.1 FIG S1 Maximum-likelihood estimates for alignmentwide mean of ω in the negative selection regime (ω ≤ 1) obtained by partitioned exploratory RELAX models. Box plots and individual points are shown side by side for each group. EF, extensive fragmenters; H, Heterotrichea; K, Karyorelictea; NEF, non-extensive fragmenters. Download FIG S1, DOCX file, 0.1 MB. Copyright © 2019 Yan et al. 2019 Yan et al. This content is distributed under the terms of the Creative Commons Attribution 4.0 International license.

10.1128/mBio.02524-19.6 TABLE S5 Relaxation/intensification parameters (*K*) for each branch group relative to NEF are shown (sorted by *P* value). Related to Table 2. Download Table S5, XLSX file, 0.03 MB. Copyright © 2019 Yan et al. 2019 Yan et al. This content is distributed under the terms of the Creative Commons Attribution 4.0 International license.

**FIG 2:**
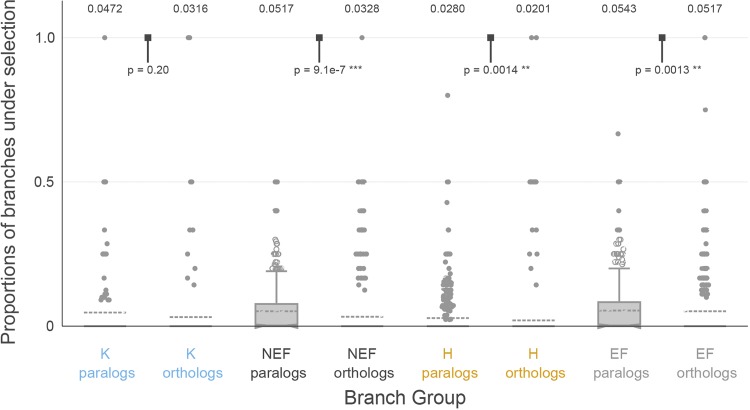
The significant difference in diversifying positive selection between paralogs and orthologs in 3 of our four focal groups estimated by aBSREL suggests that diversifying selection may occur following duplications, with Karyorelictea as the exception. The numbers above each group are group means, and *P* values are from two-sided Wilcoxon test for differences in medians. *, Significant test results.

To compare the extent to which paralogs and orthologs were subject to episodic diversifying selection, we computed, for each alignment that contained both orthologs and paralogs from the same taxonomic group, the ratio of paralog branches subject to episodic selection to the total number of paralog branches and the analogous ratio for orthologs. Comparing these fractions within a specific alignment ensures that the power to detect selection is comparable between ortholog and paralog branches. For all four taxonomic groups, a significantly higher fraction of branches was selected among paralogs than orthologs in three groups, except Karyorelictea (see Fig. 2).

### Stop codon usage and reassignment.

We assessed stop codon usage by calculating in-frame stop codon frequency, and determined amino acid reassignments by evaluating conserved sites within alignments among the diverse ciliates (see Materials and Methods for more details). We inferred a complex pattern and a considerable diversity of stop codon usage and reassignment across the ciliate phylogeny.

**FIG 3:**
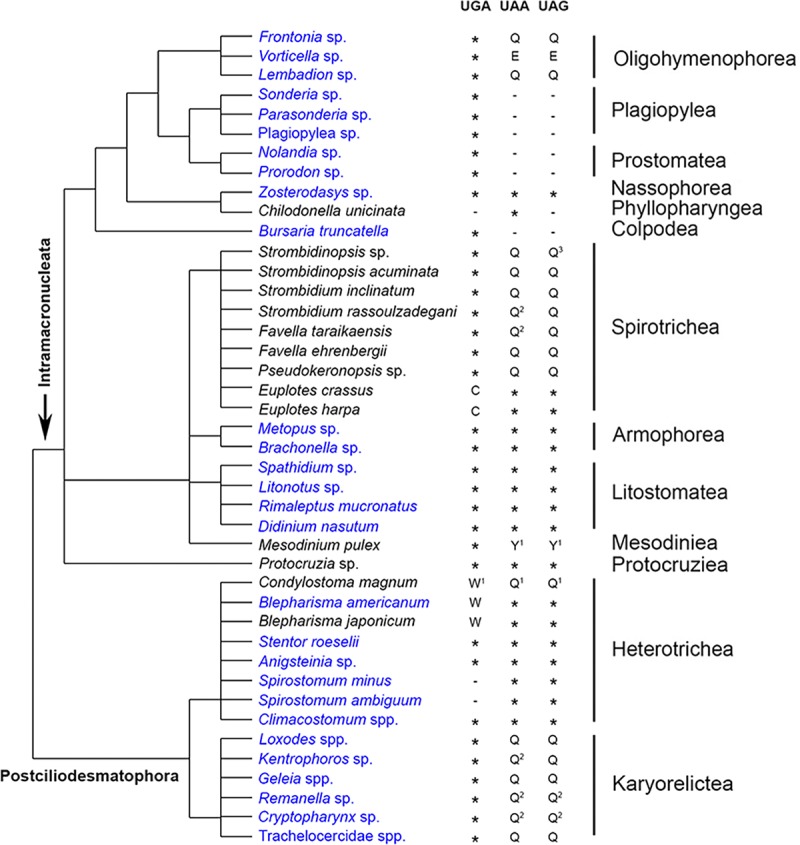
Putative stop codon usage and reassignment from single-cell transcriptomes in the present work shows variable patterns among classes. Data collected from this work are marked in blue. Phylogenetic tree topology is according to Adl et al. (61), with relationships within classes represented as polytomies; since the ciliate phylogeny is still under active debate, please see the alternative topology in Gao et al. (62). *, Serves as stop codon; –, not predominantly used as stop codon. Superscript numbers: 1, uncertainty of reassignment; 2, reassignment inferred from Swart et al. (16); 3, reassignment inferred from members from the same taxonomic group. For *Strombidinopsis* sp., we also found small and equal numbers of cases where *K* was as a reassignment for UAG.

Our data from 943 GFs shared among at least eight of the ten ciliate classes are consistent with the three major patterns previously described (15, 16) (Fig. 3): ciliates using the “universal” genetic code (UAR and UGA being stop; 11/46), those reassigning UAR to amino acids (26/46), and those that have reassigned UGA (9/46). The “ciliate” (UGA as the sole stop codon) codon table is the most common alternative genetic code in our taxonomic sampling (26/46), with the standard universal codon also being prevalent among ciliates (11/47; Fig. 3). The remaining types, the *Blepharisma* and *Euplotes* codes (UAR as stop codon; UGA coding for tryptophan and cysteine, respectively), *Chilodonella* code (UAA as stop codon; UGA and UAG coding glutamine), *Mesodinium* code (UGA as stop codon; UAR reassigned to tyrosine), and the context-dependent *Condylostoma* code, together only represent a small proportion of ciliates (9/46).

## DISCUSSION

The three main insights from this study are that (i) single-cell transcriptomics provides numerous insights into genome evolution (e.g., gene family sizes and stop codon reassignments) in uncultivable ciliates, (ii) features of genome architecture in ciliates (i.e., extensive chromosome fragmentation, high polyploidy, and paradiploidy) influence patterns of gene family evolution, and (iii) expanded taxonomic sampling reveals conservation of genetic code usage within some classes (e.g., Armophorea, Litostomatea, and Karyorelictea) and variability in others (e.g., Oligohymenophorea, Spirotrichea, and Heterotrichea). As a resource for the community, we generated single-cell transcriptomic analyses of uncultivable ciliates (Table 1) and deposited the data in the NCBI database (BioProject no. PRJNA573114; BioSample no. SAMN12807523 to SAMN12807565). These data substantially expand on the analyses of molecular evolution in ciliates by shifting the focus from cultivable model ciliates, e.g., *Tetrahymena* and *Paramecium* (44, 45), to a more comprehensive sampling of ciliate lineages.

Among the four focal clades (i.e., Karyorelictea and Heterotrichea in the Po-clade and EF and NEF in the Im-clade), the Karyorelictea possess the lowest transcript diversity (Fig. 1). This suggests that the inability of Karyorelictea to divide their somatic macronuclei (i.e., the lack of amitosis) limits GF evolution (i.e., paralog diversity); unlike other ciliates, Karyorelictea must develop a new somatic genome from the germline with every division, thus exposing any mutations accumulated in the germline in each new somatic nucleus. We speculate that this process changes fitness landscapes compared to ciliates that divide somatic nuclei by amitosis, enabling the removal of deleterious mutations through phenotypic assortment (32, 46), (i.e., stochastic distribution of alleles during somatic nuclear division). In fact, for many ciliates numerous asexual generations are necessary before sexual “maturity” (e.g., 80 to 100 generations in certain clones of Tetrahymena pyriformis [47] and 15 to 46 generations in *Euplotes crassus* [48]), during which time there is a greater opportunity to acquire potentially compensatory mutations in the germline nuclei while removing potentially deleterious mutations from their somatic nuclei (32). These compensatory mutations then appear in newly developed somatic nuclei following conjugation, allowing ciliates with amitosis to explore adaptive landscapes. Hence, the inability to undergo somatic macronuclear division (i.e., amitosis) in the Karyorelictea may explain the maintenance of fewer and less-divergent paralogs per gene family observed in this study (Fig. 1).

**FIG 4:**
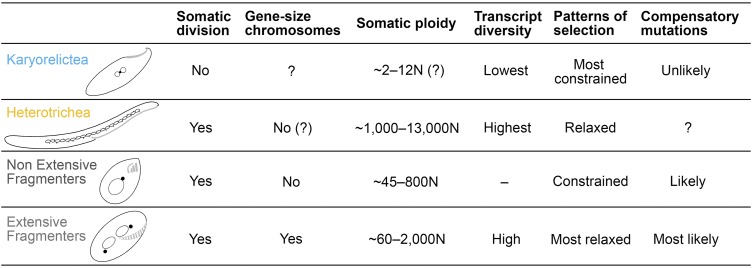
Summary of features of the four focal ciliate groups, including the ability of somatic division, somatic ploidy, the structure of somatic genome, the average transcript diversity, and patterns of selection estimated by average *dN/dS* ratio, and, based on their nuclear/genome features, how likely was it that compensatory mutations would occur in each group when mildly deleterious mutations are present. Unknown features are indicated by a question mark (“?”). Diagrams of representative members of each group are drawn with somatic nuclei in empty circles and germline nuclei in filled circles (black). Oral structures are shown in light gray.

In contrast, the Heterotrichea, the sister class of Karyorelictea, has the greatest transcript diversity among the ciliates in this study. The average number of paralogs per gene in Heterotrichea are even greater than that of ciliates with extensively fragmented somatic genomes, which are known to have large gene families composed of divergent paralogs (32, 49) (Fig. 1). All heterotrichs studied to date have extremely high somatic ploidy levels (∼1,000 to ∼13,000 N), indicating a massive amplification process during somatic macronuclear differentiation (38, 39, 50). Maurer-Alcalá et al. (51) have previously shown relatively high copy numbers of protein coding genes in *Blepharisma americanum*. If this is true for the majority of protein coding genes in heterotrichs, “errors” generated during the differentiation and amplification of a new somatic genome might contribute to the observed high transcript diversity. Intriguingly, many members in the Heterotrichea have a somatic macronucleus organized as “beads on a chain” (also observed in some other ciliate clades, e.g., Litostomatea and Spirotrichea; Fig. 4), and with only one or two beads from the somatic nucleus, *Stentor* is able to recover and regenerate itself from a partial piece (as little as 1/100th of the cell [52, 53]). At the same time, they have many germline micronuclei, e.g., 12 to 30 in *Climacostomum virens* (54) and up to 49 in *Fabrea salina* (54) (Fig. 4), and the accumulation of mutations in each individual germline nucleus might also contribute to the high transcript diversity in Heterotrichea. Further research on the physical distribution of gene copies in the nucleus is needed to assess whether there is any spatial variation in the distribution of paralogs within asexually dividing Heterotrichea somatic macronuclei.

Consistent with previous studies (32), we also detected a higher average transcript diversity in ciliates with extensively fragmented somatic genomes (the EF group) compared to the non-extensive fragmenters (the NEF group) (31, 49) (Fig. 1; *P* = 0.00153, Kruskal-Wallis non-parametric test). Our results further support the hypothesis that gene-size chromosomes enhance the rates of gene family evolution (32, 41). By breaking up gene-linkage during amitotic divisions of the somatic macronucleus, stochastic assortment of gene-sized chromosomes during amitosis is likely more efficient at purging deleterious mutations rapidly and maintaining a higher fitness that is further influenced by epigenetics (31, 55). Meanwhile, periods of sexual immaturity allow the possibility for compensatory mutations to appear in the germline, generating greater paralog diversity (32, 41) (Fig. 4).

We also estimate the selection strength among the four focal groups using the selection intensity parameter *K* and the group average *dN/dS* value. The null hypothesis is that there should be no significant difference among the four groups if genome architecture does not impact patterns of gene family evolution. To our surprise, the Karyorelictea and Heterotrichea have more gene families under intensified selection compared with NEF reference group (Table 2). Similarly, there is a trend of selection strength (measured by group average *dN/dS* values) among the four focal groups (Karyorelictea > NEF > Heterotrichea > EF; Fig. S1). The intensification and lowest *dN/dS* values suggest that Karyorelictea is under the most selective constraint, whereas the EF group is under the most relaxed selection, which could be either relaxed purifying selection or weak positive selection. Our analyses are at odds with the null hypothesis (i.e., that genome architecture and patterns of sequence evolution are not correlated) and further emphasize the impact of different genome architectures, including programmed genome rearrangements, on gene family evolution. We also tested for episodic diversifying selection between paralogs and orthologs for each group (Fig. 2). Here, a significantly higher proportion of paralogous branches under episodic selection is detected in the Heterotrichea, EF, and NEF groups, which indicates that paralogs are more likely to undergo more functional differentiation after duplication events compared to orthologs; in contrast, the generally limited number of paralogs in Karyorelictea do not show significant selective difference compared to orthologs (Fig. 2).

**TABLE 2:** Summary of 236 significant (*P* ≤ 0.05) RELAX group results among the 503 alignments tested

Selection strength	RELAX result (median *K*)^*a*^		
	Heterotrichea	Extensive fragmenters	Karyorelictea
Intensification	179 (1.51)	120 (1.36)	148 (1.53)
Relaxation	57 (0.803)	116 (0.750)	88 (0.660)

a Intensification/relaxation values for the selection for Karyorelictea, Heterotrichea, and extensive fragmenters were measured relative to non-extensive fragmenter branches. The median values of intensification/relaxation coefficients (*K*) are shown in parentheses.

We further demonstrate the diversity of patterns of stop codon usage in ciliates, and the increase in sampling shows contrasting patterns among ciliate classes. Stop codon usage appears to be conserved in some classes (e.g., in Armophorea, Litostomatea, and Karyorelictea), whereas stop codon reassignments are variable in other classes (e.g., in Heterotrichea and Spirotrichea; Fig. 3). This is consistent with previous hypotheses that mutations in the eukaryotic release factor 1 (eRF1), altering its ability to recognize certain stop codons, has evolved independently in different ciliate lineages (14–17, 56). We estimate stop codon usage in the class Karyorelictea and find all species use UGA as a stop codon, while UAR is reassigned to glutamine in *Loxodes* spp., *Geleia* spp., Trachelocercidae spp., and *Kentrophoros* sp. (the reassignment of UAA in *Kentrophoros* sp. is unclear in our data; thus, the reassignment as glutamine is inferred from other karyorelictid members). This is one of the most common types of stop codon usage patterns in ciliates, which is also found in the classes Oligohymenophorea, Colpodea, Plagiopylea, Prostomatea, Nassophorea, and Spirotrichea (UAR in *Vorticella* sp. (Oligohymenophorea) is reassigned to glutamic acid instead of glutamine, and we were unable to extract reassignments for several species due to insufficient data based on our criteria; Fig. 3). Heterotrichea remains the clade hosting the greatest diversity of genetic codes, including the extreme case, *Condylostoma magnum*, which has reassigned all three conventional stop codons and where translation termination is context dependent (15, 16). Together, these data indicate that rates of changes in stop codons are variable among ciliates, though certainly faster than other well-sampled eukaryotic clades (57).

### Synthesis.

Our analyses demonstrate the relationship between somatic macronuclear genome architecture and patterns of gene family evolution in ciliates: paralog diversity is lowest in the “paradiploid” class Karyorelictea, greater in ciliates with extensively processed genomes, and highest in the highly polyploid Heterotrichea. Similarly, there is a distinct difference in patterns of gene family evolution among those ciliates able to divide their somatic nuclei and those that cannot (i.e., Karyorelictea), which suggests that life history intersects with genome architecture in driving evolutionary patterns in ciliates. At the broadest level, our data suggest a macroevolutionary pattern, i.e., that genome architecture must be considered when developing models of molecular evolution, at least in ciliates.

## MATERIALS AND METHODS

### Sampling.

*Chilodonella uncinata*, *Blepharisma americanum*, *Rimaleptus mucronatus*, *Didinium nasutum*, and *Bursaria truncatella* were obtained from cultures, and all other taxa were collected from diverse sample sites, including a marine sandy beach, a freshwater tank, and a fen (see details in Table S1). Freshwater samples were directly poured into 5-cm petri dishes for ciliate isolation, while marine samples with sand grains were filtered through 35-μm-pore size mesh then kept in 5-cm petri dishes before single-cell transcriptome amplification.

### Single-cell transcriptomes.

Individual cells were isolated by hand using glass pipettes and washed in filtered (0.2 μm) *in situ* water three to five times prior to being placed in a minimal volume of nuclease-free water (<1 μl) in a microcentrifuge tube. Transcriptomes of the individual ciliates were generated using the SMART-Seq v4 Ultra Low Input RNA kit for sequencing (Clontech, catalog numbers 634895 and 634896) according to the manufacturer’s instructions, adjusting all measurements to a quarter reaction volume. After transcriptome amplification, we used a Nextera XT DNA library preparation kit (96 samples; Illumina, catalog no. FC-131-1096) and a Nextera XT Index kit v2 set A (96 indexes, 384 samples; Illumina, catalog no. FC-131-2001) to construct libraries for HTS. The resulting libraries were sequenced on a HiSeq 4000 (Illumina) lane at the genome sequencing center (University of California at San Diego) or at the Institute for Genome Sciences, University of Maryland, Baltimore, MD.

### Taxonomy assignment.

We collected all available small subunit (SSU) rRNA gene sequences of ciliates from the National Center for Biotechnology Information (NCBI) database and then performed BLAST searches of rRNA gene sequences isolated from each transcriptome. We inspected the top matching contigs for each cell to determine taxonomy based on identity and overlap (see the rRNA report in Table S2 and in the supplemental material).

10.1128/mBio.02524-19.3 TABLE S2 rRNA report of each transcriptome for taxonomy identification. Related to taxonomy assignment in Materials and Methods. Download Table S2, XLSX file, 0.01 MB. Copyright © 2019 Yan et al. 2019 Yan et al. This content is distributed under the terms of the Creative Commons Attribution 4.0 International license.

### Transcriptome assembly and analyses.

The output paired-end reads were trimmed with an individual quality trimming score and a minimum length of 100 bp with BBTools (58) and assembled with rnaSPAdes (part of the SPAdes v3.10.1 package [59]). After assembly, the output transcriptome was processed through a suite of custom Python scripts (part of the PhyloToL pipeline [27; https://github.com/Katzlab/PhyloTOL]). We applied PhyloToL using default settings, a relatively conservative approach, which has been successfully benchmarked in analyses of ancient eukaryotic gene families (27). The processing includes (i) the removal of contaminating rRNAs, potential mitochondrial sequences, and apparent prokaryotic transcripts; (ii) the assignment of transcripts to homologous gene families (based on the OrthoMCL database); (iii) the identification of putative ORFs from the transcripts; and (iv) the removal of transcripts of >98% nucleotide identity across ≥70% of their length to larger transcripts, which likely represent a pool of alleles, recent paralogs, and sequencing/assembly errors. The removal of potential eukaryotic contaminants was performed using outputs from the PhyloToL pipeline. For the 10 species with two (or more) available transcriptomes, we combined all transcriptomes for each species by removing partial transcripts (>98% nucleotide identity across ≥70% of length to a larger transcript) in the pool of transcriptomes (Table S1). We report average and median K-mer coverage for each data set (Table S7).

10.1128/mBio.02524-19.8 TABLE S7 Number of transcripts in each studied species in the selected 509 gene families. Related to transcript diversity analyses in Materials and Methods. EF, extensive fragmenters; NEF, non-extensive fragmenters. *, Individual cells that are combined for the analyses, respectively. Download Table S7, XLSX file, 0.2 MB. Copyright © 2019 Yan et al. 2019 Yan et al. This content is distributed under the terms of the Creative Commons Attribution 4.0 International license.

10.1128/mBio.02524-19.9 DATASET S1 SSU rRNA gene sequences from focal individuals in the present study. Download Dataset S1, TXT file, 0.1 MB. Copyright © 2019 Yan et al. 2019 Yan et al. This content is distributed under the terms of the Creative Commons Attribution 4.0 International license.

### Transcript diversity.

Together with 13 transcriptome data sets obtained from public databases (see the details in Table S3), we assessed transcript diversity in 509 GFs that contain at least one transcript in each focal clade, Karyorelictea (11 species) and Heterotrichea (8 species) in the Po-clade and extensive fragmenters (12 species) and non-extensive fragmenters (15 species) in the Im-clade (Tables S1 and S7). We counted the number of unique transcripts present in each GF for each species, and then we calculated the average transcript diversity for each clade in two ways, both including and excluding the “0” values representing the absence of transcripts in a given gene family. Here, we only show the results including the “0” values, since both analyses (with and without “0” values) are consistent (Fig. 1). To evaluate the patterns of transcript diversity, we performed boxplot analyses and Kruskal-Wallis and Mann-Whitney/Wilcoxon tests to visualize the variance among the four clades in R (42).

### Evaluating selection profiles by a phylogenetic test of selection.

We compared selection strengths between the four defined taxonomic groups (see transcript diversity) in an alignment using a group-level extension of the RELAX test (40). The test operates on a tree where branches are partitioned into *N*+1 nonoverlapping sets, where *N* sets comprise the groups of interest and the remaining set contains the “nuisance” (or unlabeled) branches. In our case, branch groups were computed as follows. Each leaf is assigned to one of the Karyorelictea (K), non-extensive fragmenters (NEF), Heterotrichea (H), and extensive fragmenters (EF) groups based on the ciliate species that it belongs to. Internal branches are labeled bottom-up (post-order tree traversal). A branch is designated as a member of K, NEF, H, or EF if and only if all of its descendant branches have the same label; otherwise, it receives no label.

RELAX models variation in selection strengths, via the ω (*dN/dS*) ratio, among sites and branches, and between groups by fitting discrete distributions to the data via maximum likelihood. Sites and branches in NEF group, which is designated as the reference group (the choice of reference should not influence test results, and NEF was chosen since it is generally the largest group, and this property facilitates numerical convergence and stability), are modeled with a 3-bin ω distribution, 0 ≤ ω_1_ ≤ ω_2_ ≤ 1 ≤ ω_3_. A *p*_1_ proportion of branch/site combinations evolve with ω_1_, *p*_2_ with ω_2_, and *p*_3_ with ω_3_ (*p*_1_ + *p*_2_ + *p*_3_ = 1). Proportions are shared among all branch groups, and ω values are scaled using the group-specific relaxation/intensification coefficient *K*[g], so that ω_g_ = ω*^K^*^[g]^. When *K*[g] > 1, all of the ω values move further away from 1 (neutrality), encoding intensification of both negative and positive selection, and when *K*[g] < 1, all of the ω values move closer to 1 (neutrality), representing relaxation of both negative and positive selection. Branches in the nuisance group are modeled with their own distribution of ω values and proportions.

The RELAX test compares the model where three values of *K*[g] are estimated from the data (one per branch group) with the model where *K*[g] = 1 (selection strength does not vary between groups). Significance is assessed via a likelihood ratio test with the χ^2^ asymptotic distribution with 3 degrees of freedom for computing *P* values. As with all group tests, this test does not identify for differences between any specified pair of groups, but rather for differences between any groups. We also fitted models where all parameters of the ω distributions were estimated separately for each branch group in order to better characterize the nature of selective processes (partitioned exploratory models [40]).

### aBSREL.

To derive branch level estimates of selective regimes, we ran the aBSREL (60) procedure on gene family alignments. This method estimates the suitable number of ω regimes for each branch, fits ω and proportion parameters, and tests, for every branch, whether or not it has evidence of ω > 1 using a likelihood ratio test.

### Assessment of stop codon usage and reassignment.

We developed custom Python scripts (https://github.com/yyan823/SCT_ciliates) to predict the in-frame stop codon usage and stop codon reassignment from each transcriptome. In brief, we collected transcripts with homologous gene family annotations and forced translation using TAA, TAG, or TGA as the only stop codon, respectively. We then calculated the frequencies of the other two traditional stop codons being in-frame. The stop codon(s) with substantial lower in-frame frequency(ies) were considered the most likely stop codon(s) for translation termination and used for further analyses. Those stop codons with heightened in-frame frequencies were then evaluated to determine their likely reassignment. For estimates of stop codon reassignments, we collected transcriptomic data from all 33 ciliate species we sampled, as well as 13 ciliate transcriptomes from the NCBI online database (https://www.ncbi.nlm.nih.gov/; Table S2), and selected 943 homologous gene families from seven well-annotated ciliate genomes (Table S4) as a reference and built alignments for each transcriptome. Conserved columns (across > 50% of the column) where stop codons were present in the taxon of question, were collected by a custom Python script (available upon request) and checked manually to calculate the frequency of the reassigned sense amino acid (Table S6).

10.1128/mBio.02524-19.5 TABLE S4 Ciliate genomes used for stop codon analyses that are obtained from public databases. Related to assessment of stop codon usage and reassignment in Materials and Methods. Download Table S4, XLSX file, 0.01 MB. Copyright © 2019 Yan et al. 2019 Yan et al. This content is distributed under the terms of the Creative Commons Attribution 4.0 International license.

10.1128/mBio.02524-19.7 TABLE S6 Counts for assessments of stop codon reassignments in ciliates that are not using conventional stop codon usage (UAR and UGA). Related to Fig. 3. Download Table S6, XLSX file, 0.01 MB. Copyright © 2019 Yan et al. 2019 Yan et al. This content is distributed under the terms of the Creative Commons Attribution 4.0 International license.

### Data availability.

Data for single-cell transcriptomic analyses of uncultivable ciliates were deposited in the NCBI database under BioProject accession number PRJNA573114 and BioSample accession numbers SAMN12807523 to SAMN12807565.
