# Supplementary material for: Single-Cell Transcriptomics Reveal a Correlation between Genome Architecture and Gene Family Evolution in Ciliates
Source: mBio. 2019 Dec 24;10(6):e02524-19. doi: 10.1128/mBio.02524-19 (PMC6935857; doi:10.1128/mBio.02524-19)
Supplement: FIG S1 [file mBio.02524-19-sf001.docx]

**Figure S1.** Maximum likelihood estimates for alignment-wide mean of ω in the negative selection regime (ω≤1) obtained by partitioned exploratory RELAX models. Box plots and individual points are shown side by side for each group. EF, extensive fragmenters; H, Heterotrichea; K, Karyorelictea; NEF, non-extensive fragementers.

**
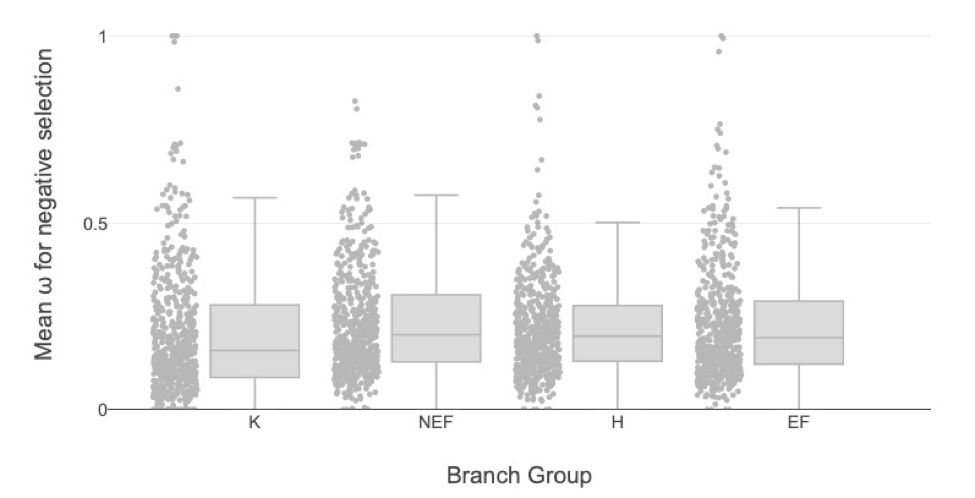
**
